# Fabrication and Performance of Self-Toughening Benzoxazine Resin and Glass Fiber-Reinforced Composites

**DOI:** 10.3390/ma19153310

**Published:** 2026-08-04

**Authors:** Yunqing Xia, Shaomu Wen, Hongfa Huang, Yanli Luo, Xu Han, Lifen Tong, Jingyu Hou, Hongjie Li

**Affiliations:** 1 Research Institute of Natural Gas Technology, Southwest Oil & Gasfield Company, Chengdu 610213, China18428301109@163.com (H.L.); 2 Southwest Oil & Gasfield Company, Chengdu 610051, China; 3 Sichuan Changning Gas Development Company, Southwest Oil & Gasfield Company, Chengdu 610051, China; 4 South Sichuan Gasfield, Southwest Oil & Gasfield Company, Luzhou 646000, China; 5 School of Materials and Energy, University of Electronic Science and Technology of China, Chengdu 610054, China; houjingyusk@163.com

**Keywords:** benzoxazine, polyarylene ether nitrile, self-toughening, composites

## Abstract

**Highlights:**

- This work pioneers the use of amino-terminated poly(arylene ether nitrile) (APEN) blended with melamine as a mixed amine source to covalently construct a self-toughening benzoxazine network
- The resin is effectively toughened without sacrificing its outstanding thermal stability or adjustable dielectric performance, while also exhibiting low water absorption.

**Abstract:**

A series of self-toughening benzoxazine resins containing amino-terminated polyarylene ether nitrile (APEN) segments were synthesized from bisphenol-A, paraformaldehyde, and a mixed amine source of APEN and melamine. Unlike conventional physical blending toughening, the APEN segments are covalently incorporated into the benzoxazine network via their amino end groups. This chemical integration not only significantly improves toughness but also simultaneously enhances thermal and dielectric properties, overcoming the common trade-off of “toughening without heat resistance”. Meanwhile, melamine serves as one of the amine sources; its excess amino groups can catalyze the ring-opening polymerization of benzoxazine, which helps to reduce the curing temperature. The effects of APEN content and curing temperature on the properties of the resin and glass fiber composites were studied. The incorporation of APEN optimized the crosslinked network, balancing rigid aromatic structures with flexible ether linkages. As the proportion of APEN segments increased, the thermal decomposition thresholds and char residue were notably enhanced, signifying progressively improved thermal resistance. For composite systems cured at 220 °C, flexural strength exhibited a continuous upward trend with rising APEN content, while the flexural modulus remained steadily within a range of 23–25 GPa, and the impact strength was remarkably elevated from 45 kJ/m^2^ to values spanning 60–73 kJ/m^2^. A further curing treatment conducted at 300 °C facilitated additional crosslinking of nitrile moieties, yielding a further enhancement in flexural strength, particularly at lower APEN contents. Fracture surface analysis confirmed the toughening effect, evidenced by the transition from smooth brittle fracture to dendritic crack patterns. In addition, the composite achieved its lowest dielectric constant of 4.2 at an APEN loading of 20 wt.% when cured at 300 °C. Overall, this investigation presented a viable and effective strategy for the design and fabrication of high-performance, self-toughened benzoxazine-based composites.

## 1. Introduction

Polybenzoxazine resins have gained significant attention as high-performance thermosetting materials because of their outstanding characteristics, which include near-zero volumetric shrinkage upon curing, excellent thermal stability, high glass transition temperatures, low water absorption, and good dielectric properties [1,2]. These attributes make them and their composites highly suitable for applications in aerospace, electronics, and advanced coatings [3,4]. However, the widespread application of benzoxazine resins is often limited by their inherent brittleness [5], a consequence of their high crosslink density and rigid aromatic backbone. This drawback results in poor fracture toughness and limits their damage tolerance, necessitating the development of effective toughening strategies that do not compromise their desirable thermal and mechanical properties [6].

Polyarylene ether nitrile (PEN) is a class of high-performance engineering thermoplastic renowned for its exceptional thermal and thermo-oxidative stability, mechanical strength, and radiation resistance, owing to the presence of aromatic rings and nitrile groups in its backbone [7,8]. Notably, the flexible ether linkages within the PEN chain impart a degree of segmental mobility, offering significant potential for toughness enhancement [9,10]. Incorporating flexible thermoplastic segments into a rigid thermosetting matrix is a well-established strategy for improving impact resistance [11,12]. Therefore, incorporating the flexible and thermally stable PEN chain segments into the benzoxazine network could be an effective approach to alleviate the inherent brittleness of polybenzoxazine while maintaining its high-temperature performance.

In this work, we report a novel approach to synthesize a benzoxazine resin integrated with PEN oligomer chains. A series of benzoxazine resins were prepared via the reaction of bisphenol-A, paraformaldehyde, and a mixed amine source comprising aminophenoxy-terminated polyarylene ether nitrile (APEN) and melamine. By co-reacting APEN with melamine, the flexible PEN segments were covalently incorporated into the crosslinked network. This study systematically investigated how the molar ratio of APEN to melamine influenced the processability, thermal properties, and mechanical performance of both the modified benzoxazine matrices and their glass fiber-reinforced composites. The findings point to establish a new technological pathway for fabricating high-temperature resistant, self-toughened benzoxazine resins and their composites.

## 2. Materials and Methods

### 2.1. Materials and Reagents

2,6-Dichlorobenzonitrile (AR), p-aminophenol (AR) were purchased from Chengdu Aoshuo Biotechnology Co., Ltd., Chengdu, China. Hydroquinone (HQ, AR), N-Methylpyrrolidone (NMP, AR) and N,N-dimethylformamide (DMF, AR) were obtained from Chengdu Kolon Co., Ltd., Chengdu, China. Anhydrous potassium carbonate (K_2_CO_3_, AR) was supplied by Chengdu Keweizhuo Technology Co., Ltd., Chengdu, China. All chemicals were used as received without further purification. The glass fiber cloth (SW210A-92a, plain weave, areal density of 210 g/m^2^) was provided by Nanjing Glass Fiber Research & Design Institute, Nanjing, China.

### 2.2. Preparation of Poly(BZ-APENn)

**Figure 1:**
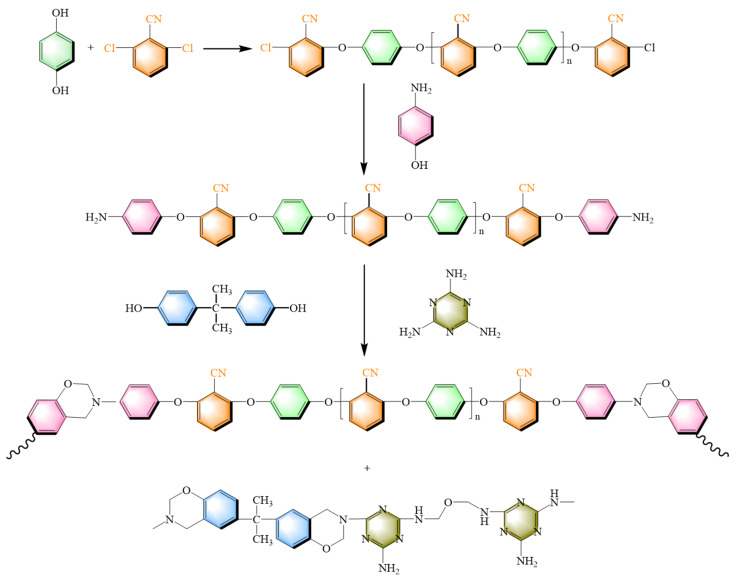
Synthetic route of BZ and BZ-APEN_n_.

The specific synthetic route was shown in Figure 1. Firstly, to fabricate APEN, anhydrous K_2_CO_3_, dichlorobenzonitrile, bisphenol A, N-methylpyrrolidone, and toluene were added to three-necked flask A at room temperature. The mixture was heated to 140–150 °C with continuous stirring and refluxed for 4–5 h. Simultaneously, 4-aminophenol, anhydrous K_2_CO_3_, and specified amounts of N-methylpyrrolidone and toluene were added to three-necked flask B at room temperature. The contents of flask B were heated to 140–150 °C using an electric heating mantle with continuous stirring and refluxed for 4–5 h. Once no significant water droplets were observed returning from the reflux, the reaction mixture from flask B was transferred into flask A and stirred for an additional 2–3 h. Upon completion, the combined mixture was poured into a beaker containing water to precipitate, yielding a dark brown solid. The precipitate was collected by filtration, dried in a vacuum oven at 80 °C, and obtained as a powder designated as APEN. The number average molecular weight (M_n_) of the APEN was determined by GPC to be 1783 g/mol with a dispersity of 1.8.

Secondly, using the APEN as one amine source and melamine as the other amine source, it reacted with bisphenol A and paraformaldehyde to synthesize benzoxazine monomers containing APEN segments (denoted as BZ-APEN_n_). The detailed synthetic procedure is as follows: N,N-Dimethylformamide was added to a flask as the solvent, followed by the introduction of paraformaldehyde, APEN, bisphenol A, and melamine. The molar ratio of phenol, amine, and aldehyde was maintained at 1:1:2. The proportions of each component in the benzoxazine formulations with varying APEN contents are listed in Table 1. The reaction mixture was stirred continuously at 80 °C for 6 h. Upon completion, the mixture was cooled to room temperature, yielding a clear and transparent brown solution. The prepared samples were designated as BZ-APEN_1_, BZ-APEN_2_, and BZ-APEN_3_, respectively. Meanwhile, a control sample, denoted as BZ, was synthesized from bisphenol A, melamine, and paraformaldehyde in N,N-dimethylformamide solvent following the same procedure.

To obtain the cured resins, the BZ-APEN monomer solutions were cast onto glass slides and subjected to a stepwise thermal curing protocol: heating at 120, 140, 160, 180, and 200 °C for 1 h each, followed by a final cure at 220 °C for 2 h. The resulting cured products were designated as poly(BZ-APEN_n_).

### 2.3. Preparation of Poly(BZ-APEN_n_)/GF

The prepared BZ-APEN_n_ monomer solution was impregnated onto glass fiber cloths (15 cm × 15 cm). The impregnated fabrics were placed in an oven at 80 °C for 30 min and then at 100 °C for 25 min to remove the solvent, forming prepregs. Subsequently, eight layers of the prepregs were stacked and compression-molded to fabricate glass fiber-reinforced composite laminates. For the laminates cured at 220 °C, the hot-pressing procedure was as follows: 140 °C for 1 h, 180 °C for 1 h, and 220 °C for 2 h. After cooling naturally to room temperature, the obtained samples were designated as poly(BZ-APEN_n_)/GF-220. Following the same procedure, composite laminates cured at 300 °C were also prepared with the hot-pressing procedure: 160 °C for 1 h, 180 °C for 1 h, 220 °C for 1 h, 240 °C for 1 h, 260 °C for 1 h, and finally 300 °C for 2 h. The resulting samples, obtained after natural cooling, were designated as poly(BZ-APEN_n_)/GF-300.

### 2.4. Characterization

The following analytical instruments were employed in the present work: a gel permeation chromatography system (Rid-20A, Shimadzu, Kyoto, Japan); a Fourier-transform infrared spectrometer (Nicolet-560, Shimadzu, Japan); a differential scanning calorimeter (Q100, TA Instruments, New Castle, DE, USA); a gelation time tester (RAY-NJ-150, Angyu Electronics, Dongguan, China); a thermogravimetric analyzer (Q50, TA Instruments, USA); a scanning electron microscope (JSM25900LV, JEOL, Tokyo, Japan); a broadband dielectric spectrometer (Concept 50, Novocontrol, Montabaur, Germany); a thermomechanical analyzer (TMA-Q400, TA Instruments, USA); a computer-controlled universal electronic testing machine (CMT6104, SANS, Shenzhen, China); a digital pendulum impact tester (XBL, Kunlun Testing Instruments, Dongguan, China). Dielectric properties were measured at a frequency of 1 kHz and room temperature (25 °C) using parallel-plate gold-sputtered electrodes (10 mm diameter) on samples with a thickness of 0.5 ± 0.1 mm. Flexural properties (ASTM D790 [13]) and unnotched Charpy impact strength (ASTM D6110 [14]) were measured on specimens of 80 × 10 × 4 mm^3^ using a span of 64 mm and a crosshead speed of 2 mm/min for flexural tests, and a hammer energy of 5 J for impact tests, with five replicates for each composition.

## 3. Results

### 3.1. Structural Characterization of BZ Monomer, APEN and BZ-APEN_n_ Monomers

**Figure 2:**
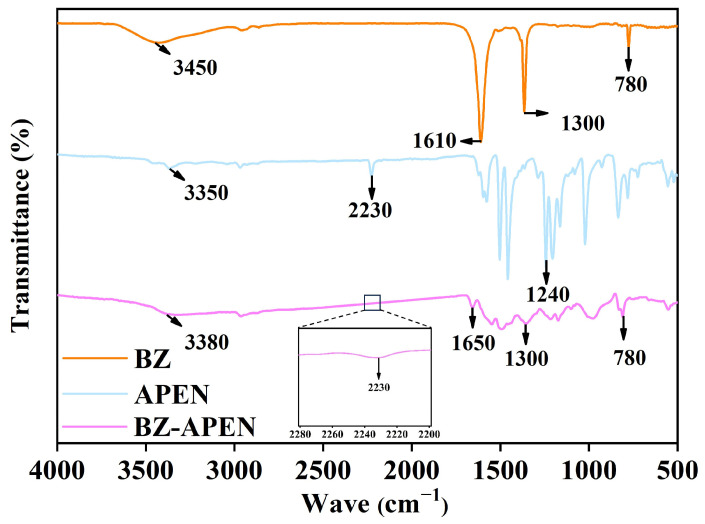
Comparative FTIR spectral profiles of BZ and APEN monomers.

To verify the successful preparation of BZ-APEN (BZ-APEN_1_ was used as the sample for the experiments), FTIR spectroscopy was employed for qualitative characterization. The comparative FTIR spectra of BZ monomer, APEN, and BZ-APEN are all presented in Figure 2. In the spectrum of the BZ monomer, an absorption band observed at 780 cm^−1^ corresponded to the characteristic signal of the benzoxazine ring, attributed to its specific vibrational mode. Additionally, the peak appearing at 1300 cm^−1^ was assigned to the asymmetric stretching vibration of the Ar–O–C linkage within the benzoxazine framework, a typical feature of the molecular backbone. The broad absorption band centered at approximately 3450 cm^−1^ and the distinct single peak observed near 1610 cm^−1^ are assigned to the N–H stretching vibration and the N–H bending vibration of the amino group in the benzoxazine structure, respectively. As for APEN, the characteristic absorption peak of cyano groups (-CN) appeared at 2230 cm^−1^. The N–H stretching vibration of primary amino groups (-NH_2_) attached to the benzene ring in APEN exhibited a single peak at 3350 cm^−1^. The asymmetric stretching vibration of aryl ether bonds (Ar–O–Ar) was observed at 1240 cm^−1^.

As observed from BZ-APEN, the absorption peaks associated with the amino functionalities of BZ-APEN underwent pronounced frequency shifts. Specifically, the broad band attributable to the N–H stretching vibration exhibited a red shift from approximately 3450 cm^−1^ to 3380 cm^−1^, whereas the band assignable to the N–H in-plane bending vibration showed a concurrent blue shift from about 1610 cm^−1^ to 1650 cm^−1^. This spectral behavior can be rationalized by the covalent bonding between the phenolic hydroxyl groups generated upon ring-opening of the oxazine moiety in BZ and the amino groups of APEN. Such spectral variations reflect the altered chemical environment of the unreacted amino moieties, arising from changes in electron cloud density and bond force constants induced by the covalent linkage. Notably, new characteristic peaks emerged after the introduction of APEN: the cyano (-CN) group exhibits a diagnostic absorption at 2230 cm^−1^, with its out-of-plane bending vibration detected at 544 cm^−1^ [15]. And the peak located at 800 cm^−1^ corresponded to the benzoxazine ring’s characteristic vibration. Additionally, the peak at 1300 cm^−1^ was assigned to the asymmetric stretching vibration of the Ar–O–C bond in the benzoxazine structure, a typical feature of the benzoxazine backbone. The new peaks confirmed the successful incorporation of cyano groups from APEN, further verifying the chemical structure of BZ-APEN_n_ monomers. The results collectively provide compelling evidence for the successful fabrication of BZ-APEN.

**Figure 3:**
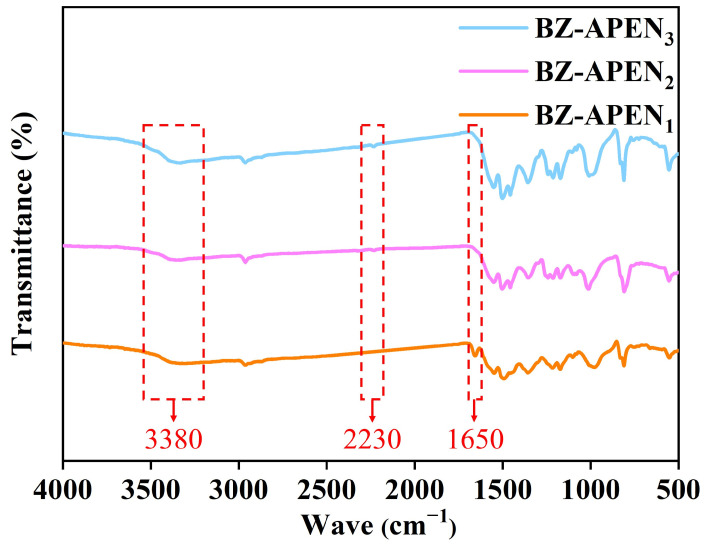
FTIR spectral profiles of BZ-APEN_n_ monomers.

A further FTIR study was conducted on BZ-APEN_n_ monomers to evaluate the effect of APEN introducing amount on the BZ-APEN. As showed in Figure 3, with increasing APEN content, the absorption peak at 1650 cm^−1^ for the BZ-APEN system exhibited a progressive attenuation, whereas the broad peak signal centered around 3380 cm^−1^ showed a gradual intensification. This trend was attributable to the enhanced ring-opening degree of the oxazine ring-conjugated structure within the benzoxazine matrix during synthesis, which is promoted by higher APEN loading. The increased extent of ring-opening accounts for the diminishing vibrational peak at 1650 cm^−1^ associated with amino groups. Concurrently, the ring-opening reaction generates phenolic hydroxyl species, and the stretching vibration of amino groups overlaps with the phenolic OH band in the 3380 cm^−1^ region. Consequently, the BZ-APEN system displays a modest signal enhancement at this wavenumber as the ring-opening progression advances. In addition, the peak intensity at 2230 cm^−1^ rose with elevating APEN proportion, which is ascribed to the growing contribution of nitrile groups originating from APEN within the hybrid system.

### 3.2. Thermal Properties of BZ-APEN_n_ Monomers

**Figure 4:**
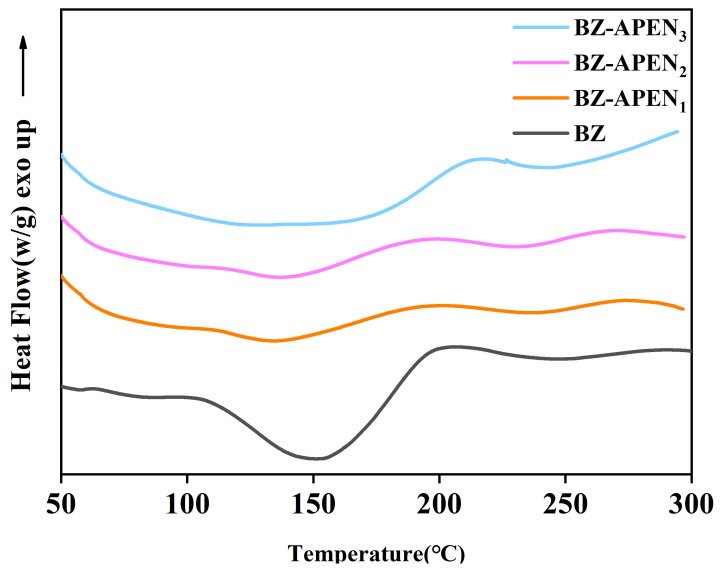
DSC thermograms of BZ and BZ-APEN_n_ monomers.

The DSC measurements of BZ and BZ-APEN_n_ monomers were conducted to verify monomers’ thermal performances (Figure 4), with relevant data summarized in Table 2. The monomers exhibited ring-opening curing peaks of benzoxazine rings in the temperature range of 160–230 °C. With increasing content of amino-terminated poly(arylene ether nitrile) segments, the initial curing temperature rose from 144.4 °C to 168.8 °C, and the curing enthalpy decreased. This phenomenon was attributed to the increased chain entanglement caused by the long-chain structure of APEN, which hindered the diffusion and movement of molecular chains during curing, leading to a shifted curing temperature. This inhibitory effect hindered the ring-opening process of benzoxazine units, thereby extending the overall reaction duration required for the monomers.

**Figure 5:**
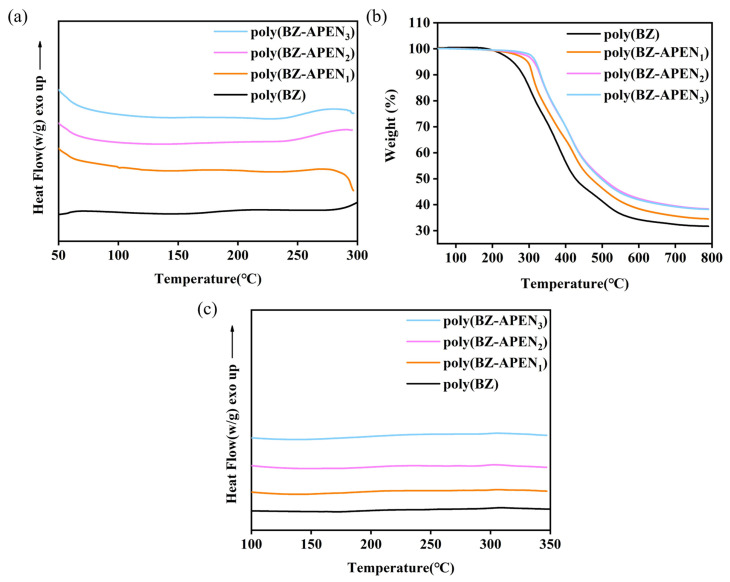
Thermal properties of poly(BZ-APEN_n_). (**a**) DSC curves at 220 °C; (**b**) TGA curves at 220 °C; (**c**) DSC curves at 300 °C.

The poly(BZ-APEN_n_)’s thermal properties were investigated through TGA and DSC tests, depicted as spectra curves in Figure 5. The detail parameters are displayed in Table 3. As shown in Figure 5a and Table 3, almost no obvious curing enthalpy was detected in the range of 150–200 °C for resins cured at 220 °C, indicating that the ring-opening reaction of oxazine rings was nearly completed in this temperature interval. The curing temperature of 220 °C triggered the ring-opening of most oxazine rings and the formation of cross-linked structures, resulting in no significant thermal effect in this range. However, a certain curing enthalpy was still observed in the high-temperature region of 250–300 °C in the DSC curves, which might correspond to the curing peak of cyano groups [16]. This was ascribed to the increased molecular chain segments and the introduction of -CN groups, which significantly enhanced the cross-linking density.

The TGA curves of cured resins at 220 °C (Figure 5b) revealed that the 5% and 10% weight loss temperatures (T_d5%_ and T_d10%_) and char yield (Y_c_) were remarkably improved with increasing APEN content. This was mainly due to the rigid aromatic ring structure of amino-terminated poly(arylene ether nitrile) and its promotion effect on the cross-linking behavior of the resin. The abundant aromatic rings in poly(arylene ether nitrile) endowed excellent thermal stability and effectively improved the overall heat resistance of the resin. The increased proportion of rigid aromatic rings suppressed the thermal motion of molecular chains and delayed thermal decomposition, leading to elevated decomposition temperatures. Moreover, the introduction of poly(arylene ether nitrile) facilitated the formation of a cross-linked network, and the increased cross-linking density further enhanced the thermal stability of the resin.

**Figure 6:**
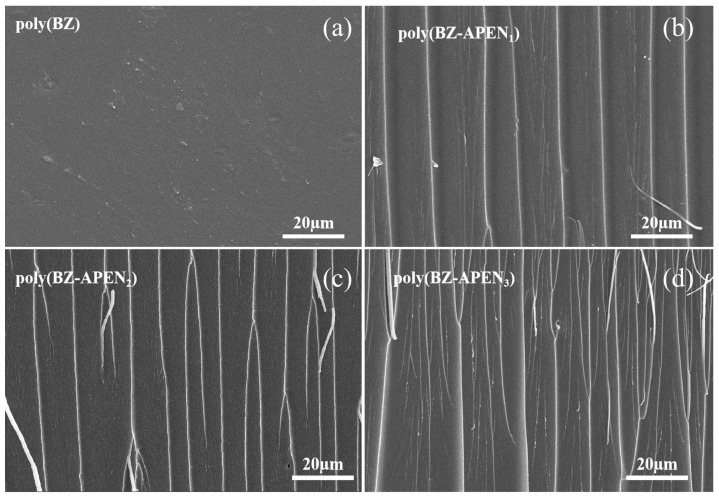
Cross-sectional SEM images of polymer films. (**a**) poly(BZ); (**b**) poly(BZ-APEN_1_); (**c**) poly(BZ-APEN_2_); (**d**) poly(BZ-APEN_3_).

According to Equation (1), the curing degrees of samples cured at 220 °C are 60%, 78%, 86%, 86%, respectively. As illustrated in Figure 6c, the curing reaction of all resins was nearly completed when the curing temperature reached 300 °C, demonstrating the excellent curing efficiency of the system at high temperatures.

Curing Degree (%) = (1 − ΔH/ΔH_0_) × 100% (1)

where

ΔH_0_ = total heat of reaction for a fully cured sample (J/g), which is the enthalpy released when the material cures completely from an uncured state. In this system, this value is listed in Table 2.

ΔH = residual heat of reaction remaining in a partially cured sample (J/g), which is listed in Table 3.

### 3.3. Morphological Analysis of Poly(BZ-APEN_n_)s

Figure 6 shows the SEM fracture surfaces of benzoxazine resin films cured at 220 °C, highlighting the marked effect of APEN incorporation. Without APEN, the fracture surface was smooth and brittle, due to the rigid, low-toughness molecular chains, easy crack propagation along stress concentrations. With APEN (Figure 6b–d), the morphology shifted from smooth to vertical stripe patterns and dendritic cracks, whose density increased with APEN content. This change resulted from the flexible segments of APEN, which enhanced toughness, dissipated energy via chain extension and slippage, and hindered rapid crack growth. Meanwhile, the rigid aromatic rings of APEN interacted strongly with those of the benzoxazine resin, further improving mechanical performance. Consequently, the material fractured with greater toughness, producing more tortuous crack paths, visible as vertical stripes and dendritic cracks.

### 3.4. Water Absorption of Poly(BZ-APEN_n_) Resins

**Figure 7:**
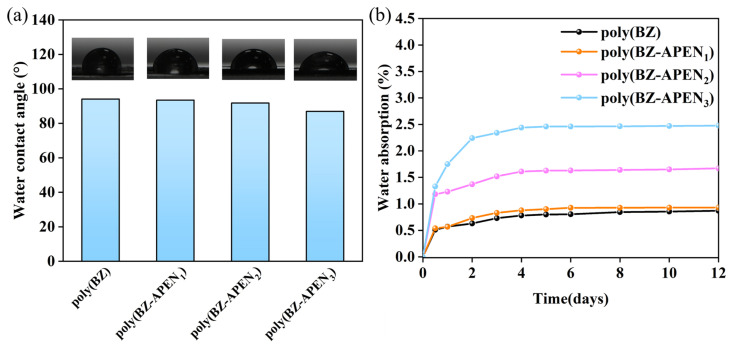
Water absorption properties of poly(BZ-APEN_n_). (**a**) Water contact angles; (**b**) Water absorption versus time curves.

Figure 7a showed the water contact angles on the surfaces of cured films. The water contact angle gradually decreased with increasing amino-terminated poly(arylene ether nitrile) content, indicating enhanced hydrophilicity. APEN contained polar groups such as amino (-NH_2_) and cyano (-CN) groups, which were highly hydrophilic. The increased number of polar groups on the resin surface raised the surface energy. As polar molecules, water molecules easily formed hydrogen bonds with amino and cyano groups on the resin surface, enhancing hydrophilicity.

The water absorption of polymer films stored at room temperature for 12 days is presented in Figure 7b. The water absorption continuously increased with immersion time and reached equilibrium after 10 days. Meanwhile, the water absorption of poly(BZ-APEN_n_) gradually rose with increasing APEN loading. When the APEN content was 10 wt.%, the equilibrium water absorption of poly(BZ-APEN_1_) after 12 days was 0.7%, higher than that of poly(BZ). This was caused by the abundant polar groups in poly(arylene ether nitrile).

### 3.5. Characterization of Poly(BZ-APEN_n_)/GF

#### 3.5.1. Thermal Expansion Properties of Poly(BZ-APEN_n_)/GF

**Figure 8:**
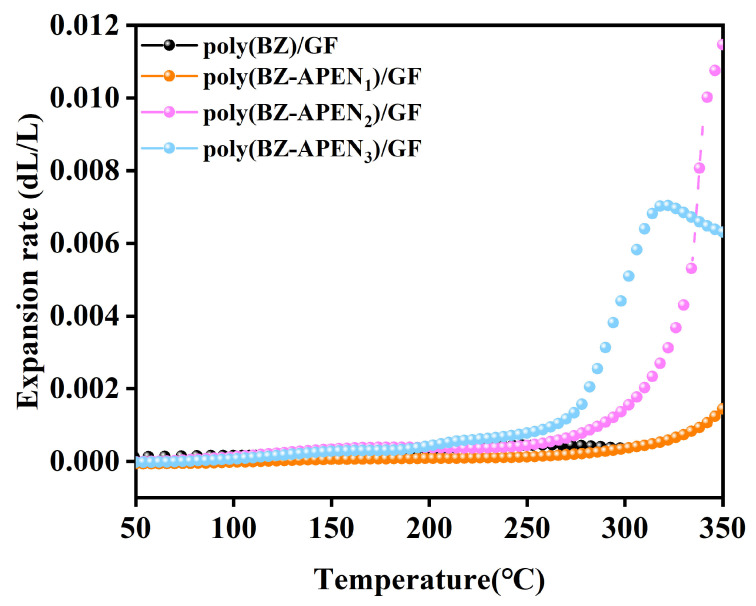
TMA profiles of poly(BZ)/GF and poly(BZ-APEN_n_)/GF composites.

To further expand the application potential of melamine-based benzoxazine resins, glass fiber-reinforced laminate composites were fabricated using poly(BZ-APEN_n_) as the polymer matrix, and their thermal resistance and mechanical properties were systematically evaluated. These composites exhibited outstanding performance over a wide temperature range, particularly in terms of the coefficient of thermal expansion (CTE), which was calculated from the slope of the dimension change vs. temperature curve over the range of 50–250 °C (below the glass transition). As shown in Figure 8, the composites demonstrated a low CTE along the *Z*-axis, reaching 23 ppm/°C when the APEN content was 20 wt.%. This low CTE indicated excellent dimensional stability under high-temperature conditions, effectively resisting deformation caused by temperature fluctuations. Furthermore, the glass transition temperature (T_g_, which was determined from the peak of the derivative of the TMA curve corresponding to the CTE change) of the composites varied markedly with the introduction of different APEN loadings, which was taken as the onset temperature of the sharp dimensional change in the TMA curve (i.e., the intersection of the baseline and the tangent of the transition region). Specifically, T_g_ initially increased and then decreased. An appropriate amount of APEN, acting as rigid segments within the crosslinked network, restricted chain mobility through its aromatic ring structures and synergistically enhanced the crosslinking density, raising T_g_ to 270 °C. However, excessive APEN led to aggregation and macroscopic phase separation, disrupting the continuity of the crosslinked network. Concurrently, the relaxation effect of the thermoplastic segments became more pronounced, causing an overall decline in T_g_. In addition, the reduced compatibility between high APEN content and the matrix induced microphase separation, forming interfaces between APEN-rich domains and the matrix. Weak bonding regions at these phase boundaries underwent preferential chain segmental motion during heating, further lowering the composite’s T_g_. Notably, an excess of APEN may also introduce network defects due to uneven crosslinking density distribution, which acted as activation sites for segmental motion upon thermal stimulation, thereby accelerating the glass transition process. Overall, the glass fiber-reinforced laminate composites based on the poly(BZ-APEN_n_) matrix exhibited excellent thermal and mechanical performance. Their low *Z*-axis CTE and high T_g_ offered significant advantages for high-temperature applications, particularly in fields demanding high dimensional stability and thermal resistance, such as aerospace, electronic packaging, and high-temperature structural materials. These remarkable properties provided a crucial theoretical and practical foundation for further development of melamine-based benzoxazine resins.

#### 3.5.2. Mechanical Properties of Poly(BZ-APENn)/GF

**Figure 9:**
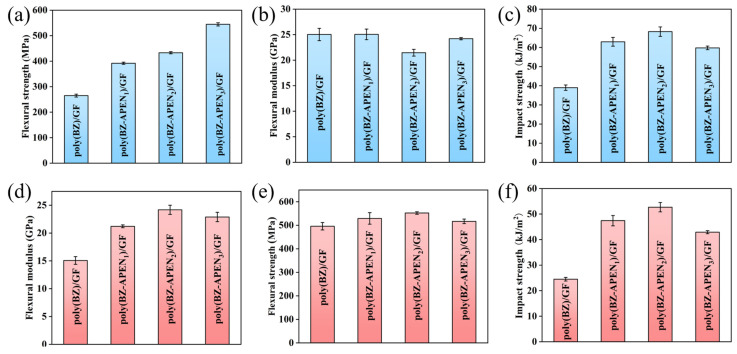
Mechanical properties of poly(BZ)/GF and poly(BZ-APEN_n_)/GF composites. (**a**–**c**) Samples cured at 220 °C; (**d**–**f**) Samples cured at 300 °C.

Figure 9 summarizes the mechanical behavior of poly(BZ)/GF and poly(BZ-APEN_n_)/GF composite laminates fabricated at 220 °C and 300 °C. As delineated in Figure 9a, the flexural strength exhibited a pronounced positive correlation with APEN content. This observation was rationally ascribed to the progressively established, densely interwoven crosslinking network arising from the concurrent reactions of benzoxazine rings and cyano groups as the APEN loading increases. Notably, Figure 9d revealed a further elevation in flexural strength at 300 °C, which was predominantly attributable to the additional crosslinking of cyano moieties under high-temperature conditions. This process not only markedly diminished structural imperfections such as voids or unreacted zones within the material but also enhanced the rigidity of molecular chains via the conjugative effect of rigid aromatic rings, thereby synergistically advancing the flexural performance. The impact strength of the as-prepared composite laminates served as a direct and sensitive indicator of the toughening efficacy imparted by APEN to the benzoxazine matrix. As evidenced by Figure 9c, the integration of APEN significantly elevated the impact strength from 45 kJ/m^2^ to an enhanced range spanning 60–73 kJ/m^2^. Among the series, poly(BZ-APEN_2_) demonstrated a comparatively superior impact resistance. This remarkable improvement was predominantly attributed to the high intrinsic rotational freedom characteristic of the ether linkages within APEN, coupled with the dynamic segmental interactions along its backbone. Such molecular featured effectively dissipate localized stress concentrations within the material, thereby yielding a pronounced toughening effect.

As the temperature rose (depicted in Figure 9f), the elevated thermal condition accelerated both the ring-opening polymerization of benzoxazine and the crosslinking reactions among APEN segments, thereby fostering the formation of a highly compact three-dimensional network. Such excessive crosslinking inevitably restricted the localized segmental mobility of the polymer chains, suppressing plastic deformation and energy dissipation, which in turn led to a discernible reduction in the impact strength of the material. The flexural modulus of the composites fabricated at 220 °C is presented in Figure 9b, where it remained stable within the range of 23–25 GPa. This stability stemmed from a dynamic equilibrium between rigid aromatic rings and flexible chain segments: the rigid structural components dominated the overall framework stiffness, while the flexible segments mitigated the brittleness otherwise induced by excessive crosslinking. The synergistic interplay between these two structural motifs effectively confined the flexural modulus to the narrow window of 23–25 GPa. However, upon further elevation of the temperature, as shown in Figure 9e, excessive crosslinking may render the network excessively rigid, thereby limiting the local mobility of molecular chains. This rigidity impeded the material’s ability to dissipate stress through microdomain deformation under low-strain conditions, ultimately resulting in a moderate decline in flexural modulus.

#### 3.5.3. Dielectric Properties of Poly(BZ-APEN_n_)/GF

**Figure 10:**
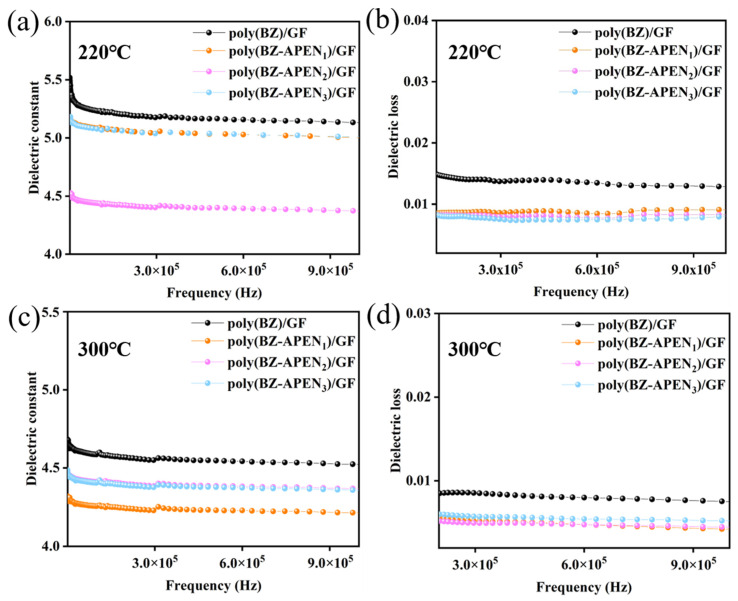
Dielectric properties of poly(BZ)/GF and poly(BZ-APENn)/GF composites. (**a**) dielectric constant at 220 °C; (**b**) dielectric loss at 220 °C; (**c**) dielectric constant at 300 °C; (**d**) dielectric loss at 300 °C.

Figure 10a,b show the dielectric properties of the composites prepared at 220 °C. With increasing APEN content, dielectric constant first decreases (minimum 4.4 at 20 wt.%) then increases, while dielectric loss continuously drops. The initial decrease is due to APEN’s rigid aromatic rings restricting dipole motion. The later increase in dielectric constant results from more cyano groups enhancing intrinsic polarization and excess APEN inducing microphase separation with interfacial polarization. Loss keeps decreasing because the rigid structure persistently suppresses chain relaxation.

When the curing temperature is raised to 300 °C, as shown in Figure 10c,d, dielectric constant shows a similar trend but reaches a minimum of 4.2 at 10 wt.% APEN; loss first decreases (lowest at 20 wt.%) then increases. High-temperature curing accelerates cyano crosslinking, forming a dense network that immobilizes polar groups and restricts dipoles, hence the lower APEN content for minimum ε′. Beyond that, increased APEN worsens compatibility, causing microphase separation and exposed unreacted polar groups, raising ε′. For loss, rigid rings initially reduce it, but above 20 wt.% phase separation and unreacted polar groups enhance interfacial loss and dipole flipping, turning loss upward.

## 4. Conclusions

This work demonstrated that amino-terminated poly(arylene ether nitrile) (APEN) serves as an effective modifier for benzoxazine composites, offering synergistic improvements in mechanical performance, thermal stability, water resistance, and dielectric properties. By adjusting the melamine/APEN ratio, the processing window of benzoxazine was broadened, while optimized APEN incorporation reduced the coefficient of thermal expansion and rose the glass transition temperature, though excessive loading triggers phase separation and property degradation. Composites with moderate APEN content exhibited significantly enhanced flexural strength (modulus maintained at 23–25 GPa) and impact strength, alongside a low equilibrium water absorption of 0.7% and tunable dielectric behavior, providing a viable strategy for designing high-performance benzoxazine-based materials.

## Figures and Tables

**Table 1:** Component ratios in BZ-APEN_n_ with different APEN contents.

Sample	Bisphenol A (mol)	APEN (mol/g)	Melamine (mol/g)	Paraformaldehyde (wt.%)	APEN Loading (wt.%) Against Nitrogen Source Compound
BZ-APEN_1_	0.2067	0.0016/2.8	0.2051/25.8	0.8267	10
BZ-APEN_2_	0.1992	0.0034/6.1	0.1958/24.6	0.7967	20
BZ-APEN_3_	0.1917	0.0056/10.0	0.1861/23.4	0.7667	30

**Table 2:** Thermal properties of BZ and BZ-APEN_n_ monomers.

Sample	ΔH (J/g)	T_i_ (°C)	T_p_ (°C)
BZ	47.2	144.4	201.5
BZ-APEN_1_	48.0	150.5	191.6
BZ-APEN_2_	45.8	151.6	192.2
BZ-APEN_3_	30.4	168.8	209.1

**Table 3:** Thermal properties of poly(BZ) and poly(BZ-APENn).

Sample	ΔH (J/g)	Td_5%_ (°C)	Td_10%_ (°C)	Yc (%)
poly(BZ)	18.9	288.9	309.1	36.2
poly(BZ-APEN_1_)	10.5	305.8	310.6	39.9
poly(BZ-APEN_2_)	6.2	323.7	333.8	42.4
poly(BZ-APEN_3_)	4.1	323.9	333.9	42.5

## Data Availability

The original contributions presented in this study are included in the article. Further inquiries can be directed to the corresponding authors.
